# Clinical Value of Fecal Calprotectin in Predicting Mucosal Healing in Patients With Ulcerative Colitis

**DOI:** 10.3389/fmed.2021.679264

**Published:** 2021-08-03

**Authors:** Fang Chen, Yue Hu, Yi-Hong Fan, Bin Lv

**Affiliations:** ^1^Department of Gastroenterology, Zhejiang Hospital of Integrated Traditional Chinese and Western Medicine, Hangzhou, China; ^2^Department of Gastroenterology, The First Affiliated Hospital of Zhejiang Chinese Medical University, Hangzhou, China

**Keywords:** biomarkers, ulcerative colitis, fecal calprotectin, mucosal healing, clinical value

## Abstract

**Aim:** This study aimed to evaluate the clinical significance of fecal calprotectin (FC) in assessment of ulcerative colitis (UC) patients' endoscopic patterns and clinical manifestation.

**Methods:** A total of 143 UC patients who received colonoscopy and 108 controls were included. After providing stool samples, patients underwent total colonoscopy. FC was measured by an enzyme-linked immunosorbent assay (ELISA). Clinical activity was based on the Mayo score. Endoscopic findings was scored by the Ulcerative Colitis Endoscopic Index of Severity (UCEIS). Correlation analysis and receiver-operator characteristic (ROC) analysis were carried out to determine the significance of measurements.

**Results:** The median (interquartile range, IQR) of FC levels was 211 (43–990) μg/g in UC and 87.5 (40.50~181) μg/g in the control group. Fecal calprotectin correlated significantly with both Mayo and UCEIS scores (Spearman's r 0.670 and 0.592, *P* < 0.01). With a cut-off value of 164 μg/g for fecal calprotectin concentration, the area under the curve (AUC) in receiver operator characteristic analysis was 0.830, sensitivity was 85.42%, specificity was 73.68%, positive predictive value (PPV) was 62.12%, and negative predictive value (NPV) was 9.10% in predicting clinical active disease. Similarly, the power of FC to predict mucosal healing (MH) was modest. With a cut-off value of 154.5 μg/g, the AUC was 0.839, sensitivity was 72.34%, and specificity was 85.71%.

**Conclusion:** For evaluating the disease activity of UC, FC is a clinically relevant biomarker for both clinically active disease and MH in patients with UC. But the cut-off value still needs large and multicenter studies for confirmation.

## Introduction

Ulcerative colitis (UC) is a chronic disease with a remitting and relapsing course. For evaluation of disease course and for monitoring treatment response, reliable tools are essential. Assessment of UC activity in clinic is usually based on a combination of clinical manifestations and laboratory tests. The current gold standard is colonoscopy because symptoms do not precisely reflect intestinal inflammation and mucosal healing (1). Endoscopic procedures, however, are unpleasant, sometimes painful, and time-consuming in China. Fecal calprotectin (FC) is a calcium-binding, cytosolic protein in neutrophils which has antimicrobial and antiproliferative properties. Fecal calprotectin concentration reflects the increased migration of neutrophils through the inflamed bowel wall to the lumen (2). In stool, calprotectin is degradation-resistant, stable, and easily measurable by ELISA (3). The test has been used successfully to distinguish inflammatory from functional bowel disorders (4). Recent studies suggested that FC levels correlate well with endoscopic indices of UC activity including Matts' index (5), Sutherland criteria (6), Rachmilewitz index (7), and the Mayo endoscopic subscore (8). In addition, elevated FC may indicate an increased risk of disease relapse (9, 10).

Since longstanding active inflammation is also considered a risk factor for the development of tissue destruction, dysplasia, and cancer (11), healing of the mucosa may also lead to a reduction in those complications. For these reasons, mucosal healing has been brought into the treat to target era. The current study found that a subgroup of patients had persistently active endoscopic inflammation while in clinical remission (12). Obviously, a noninvasive biomarker to identify patients with MH is preferable in clinical settings. This could allow more regular assessment of inflammation and possibly lead to a reduced requirement for follow-up endoscopies.

In recent years, various biomarkers of MH have been explored such as C-reactive protein (CRP) and erythrocyte sedimentation rate (ESR). Because in UC patients, inflammation is mainly confined to the colon and the rectum, it may be reasonable that a fecal marker is more accurate than a serum marker.

The aim of this study was to evaluate the clinical significance of FC in the assessment of UC clinical activity and MH. Additionally, cut-off levels were also determined for the clinical activity and MH.

## Methods

### Patients

A total of 143 adult outpatients and inpatients with a previously confirmed diagnosis of UC referred for colonoscopy at the Departments of Gastroenterology of the First Affiliated Hospital of Zhejiang Chinese Medical University between May 2015 and December 2016 were included. They were diagnosed on the basis of clinical, endoscopic, and histologic criteria. A second cohort of 108 healthy volunteers served as controls. The disease extension was classified according to the Montreal classification (13). Exclusion criteria included pregnancy, colorectal cancer, history of bowel resection, long-term use of NSAIDs, or presence of comorbidities that could cause inflammatory reactions, active infection, incomplete colonoscopy (not reaching the cecum), and inability to provide stool samples.

Clinical disease severity was assessed according to Mayo scores. Clinical disease activity was divided into clinical remission (0–2), mild (3–5), moderate (6–10), and severe (11–12) according to the frequency of defecation, hematochezia, and findings of colonoscopy and physician's global assessment. The UCEIS score (14, 15), composed of vascular pattern (0–2), bleeding (0–3), and erosions and ulcers (0–3), was applied to evaluate endoscopic activity, while MH (16) was defined as UCEIS 0 or 1, and UCEIS 1 was limited to vascular patterns.

### Study Protocol

Patients provided stool samples within the previous 7 days of the colonoscopy (prior to bowel preparation), and the stool samples were stored at −20°C until assay. After bowel preparation, patients underwent total colonoscopy, and UCEIS score was used to assess MH. The greatest score in any anatomical site was recorded.

### Fecal Calprotectin Assays

Stools were collected within the previous 7 days of the colonoscopy, and immediately stored at −20°C. The stool samples were sent to Suzhou Herui IBD Project Center, and fecal calprotectin was measured in a blind manner using the PhiCal enzyme-linked immunosorbent assay (ELISA) Assay.

### Statistics

For numerical variables, median and interquartile range (IQR) were calculated, and the Mann–Whitney *U*-test was applied. The Spearman correlation analysis between FC and clinical/endoscopic disease severity was carried out. The best cut-off for FC to predict clinical activity and MH were calculated by using receiver–operator characteristic (ROC) graphs. According to the cut-off levels, test significance including sensitivity (SENS), specificity (SPEC), positive–predictive value (PPV), negative predictive value (NPV), and accuracy rate (AR) were calculated. Two sided *P* < 0.05 were considered to be statistically significant.

## Results

### Characteristics of the Participants

Overall, 143 UC patients and 108 controls were included in the study. Among the 143 UC patients (44% women), the mean age at the time of inclusion was 43.64 ± 13.62 years. While ulcerative colitis extent was limited to the rectum in 52 patients (36.36%), 27 patients (18.88%) had sigmoid/left colon involvement and 44 patients (30.77%) had pancolitis. Patients' characteristics are shown in Table 1. According to Mayo scores, 49 (34.27%) patients were in remission, 46 (32.17%) patients had mild, 41 (28.67%) patients had moderate, and 7 (4.90%) patients had severe disease activity. Overall, mucosal healing, defined as UCEIS score 0 or 1, was observed in 48 ulcerative colitis patients (33.57%).

**Table 1:** Baseline demographic variables of patients included in this study.

	**UC**	**Control**
N	143	108
Male/female	80/60	47/61
Age (Mean ± SD)	43.64 ± 13.62	48.53 ± 16.30
**Age at diagnosis (years)**
A1 (≤16)	0	
A2 (17–40)	88 (61.54%)	
A3 (≥40)	55 (38.46%)	
**Disease location**
Non	20 (13.99%)	
E1	52 (36.36%)	
E2	27 (18.88%)	
E3	44 (30.77%)	
**Mayo grades**
Remission (≤2)	49 (34.27%)	
Mild activity (3–5)	46 (32.17%)	
Moderate activity (6–10)	41 (28.67%)	
Severe activity (11–12)	7 (4.90%)	

**Figure 1:**
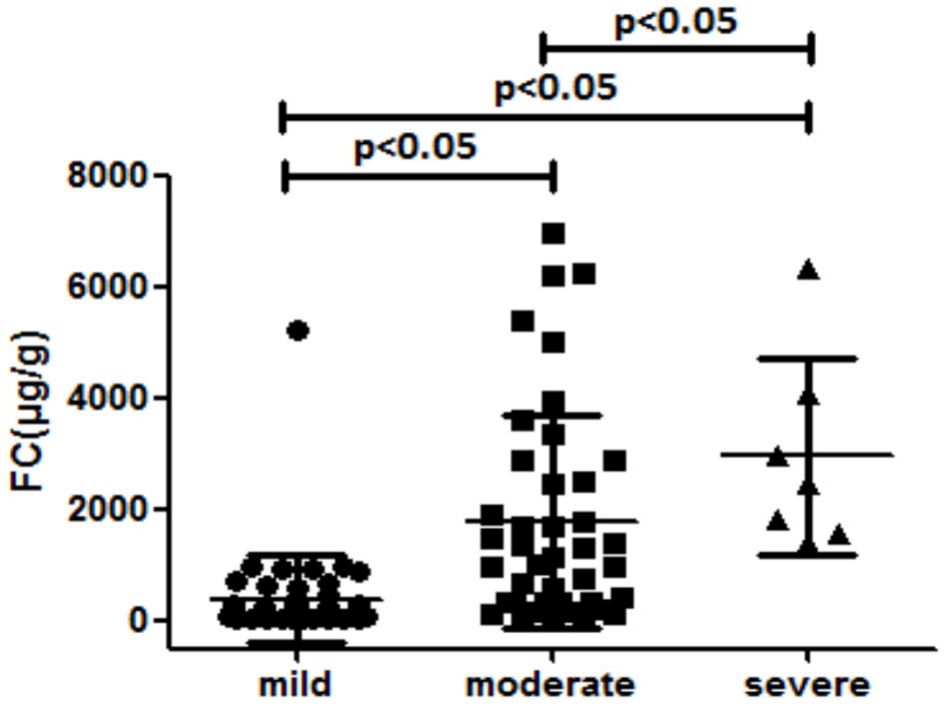
Median fecal calprotectin levels (interquartile range) in patients stratified according to the Mayo grades (μg/g).

In total, 108 controls were studied (56% women). Their median age was 48.53 ± 16.30 years. The median fecal calprotectin in this group was 87.5 (IQR 40.50–181) μg/g. The median (IQR) value for FC level of all patients was 211 (43–990) μg/g. There was a significant difference in the FC concentration between the UC and the controls (*P* < 0.05; Table 2). The FC concentration were 38 (30–102.5) μg/g, 220.5 (87–367.75) μg/g, 1,138 (340.50–2699) μg/g, and 2,481 (1573–4067) μg/g, respectively with each stage classified by Mayo scores. As seen in Figure 1 and Table 2, there was a significant difference in FC levels between patients with mild disease and moderate disease (*P* < 0.05) as well as between moderate disease and severe disease (*P* < 0.05).

**Table 2:** Median fecal calprotectin levels (interquartile range) in patients stratified according to the Mayo grades (μg/g).

**Variable**	**N**	**FC (μg/g)**			
		**Median**	**Quartile**	**Min**~**Max**	**Interquartile range (IQR)**
Control	108	87.5	141	11~1,560	40.50~181
UC	143	211	947	17~6,964	43~990
Remission	49	38^*^	73	22~5,321	30~102.5
Mild activity	46	220.5	281	17~5,235	87~367.75
Moderate activity	41	1,138Δ	2359	26~6,964	340.50~2,699
Severe activity	7	2,481ΔΔ	2494	1,414~6,324	1,573~4,067

* *p < 0.05 (p = 0.002), vs. the control; Δp < 0.05 (p = 0.000), vs. the mild group; ΔΔp < 0.05 (p = 0.000, p = 0.033, respectively), vs. the mild and moderate group*.

### Correlation Analysis

**Figure 2:**
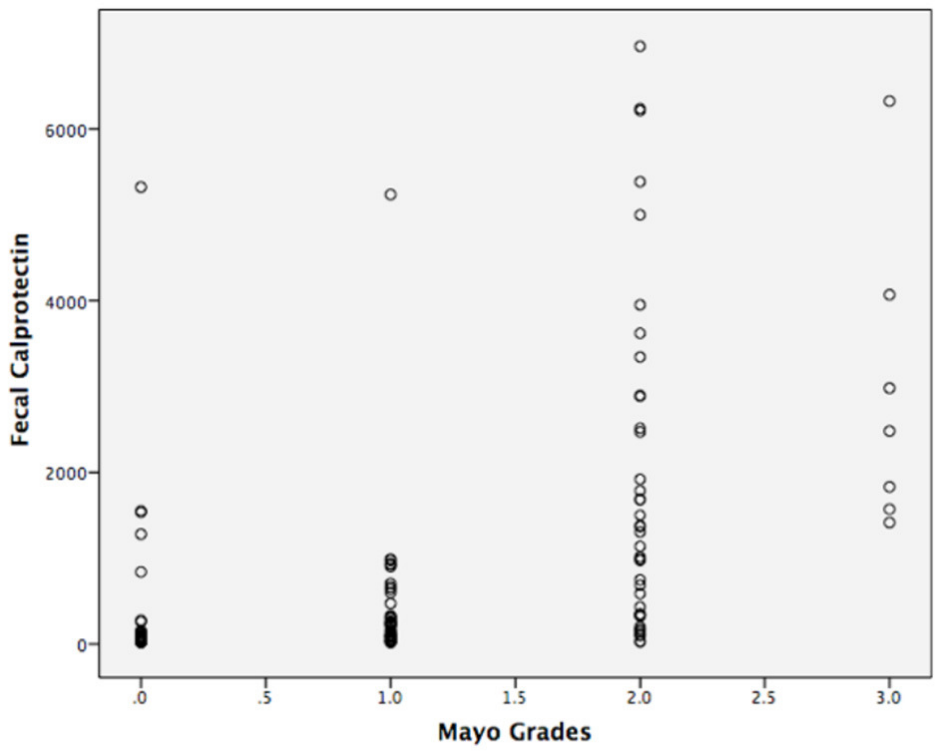
Concentrations of fecal calprotectin and the Mayo grades of UC (*r* = 0.670, *p* < 0.01).

**Figure 3:**
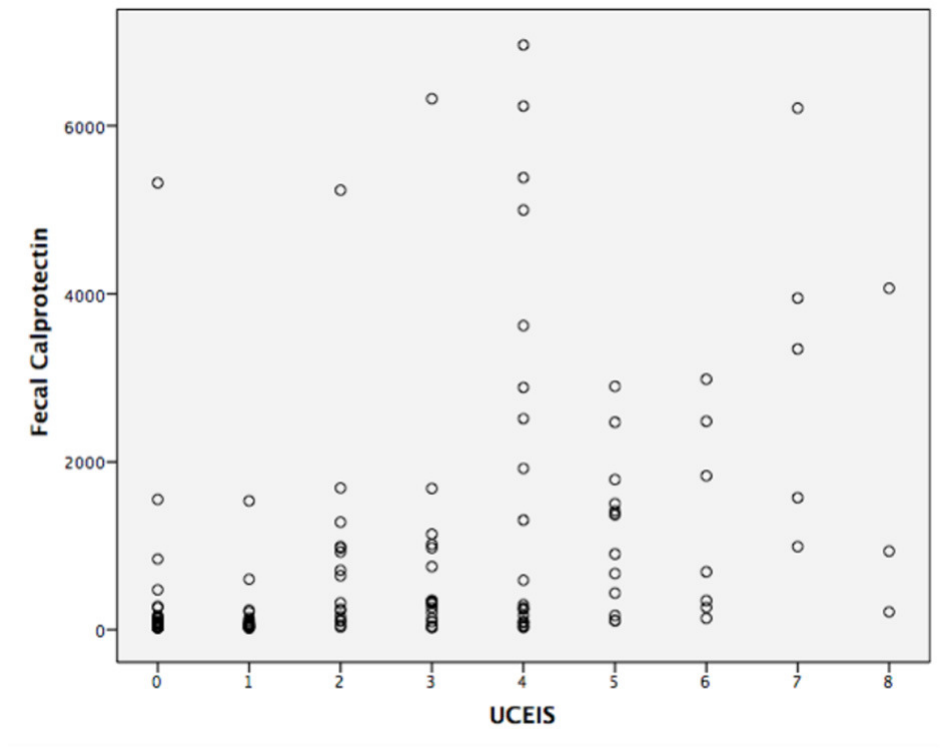
Concentrations of fecal calprotectin and the UCEIS scores of UC (*r* = 0.670, *p* < 0.01).

The correlation analysis is shown in Figures 2, 3. The Mayo grades and the UCEIS scores both correlated very well with the FC levels (*r* = 0.670, *P* < 0.01, and *r* = 0.592, *P* < 0.01, respectively).

### ROC Curve Analysis

**Figure 4:**
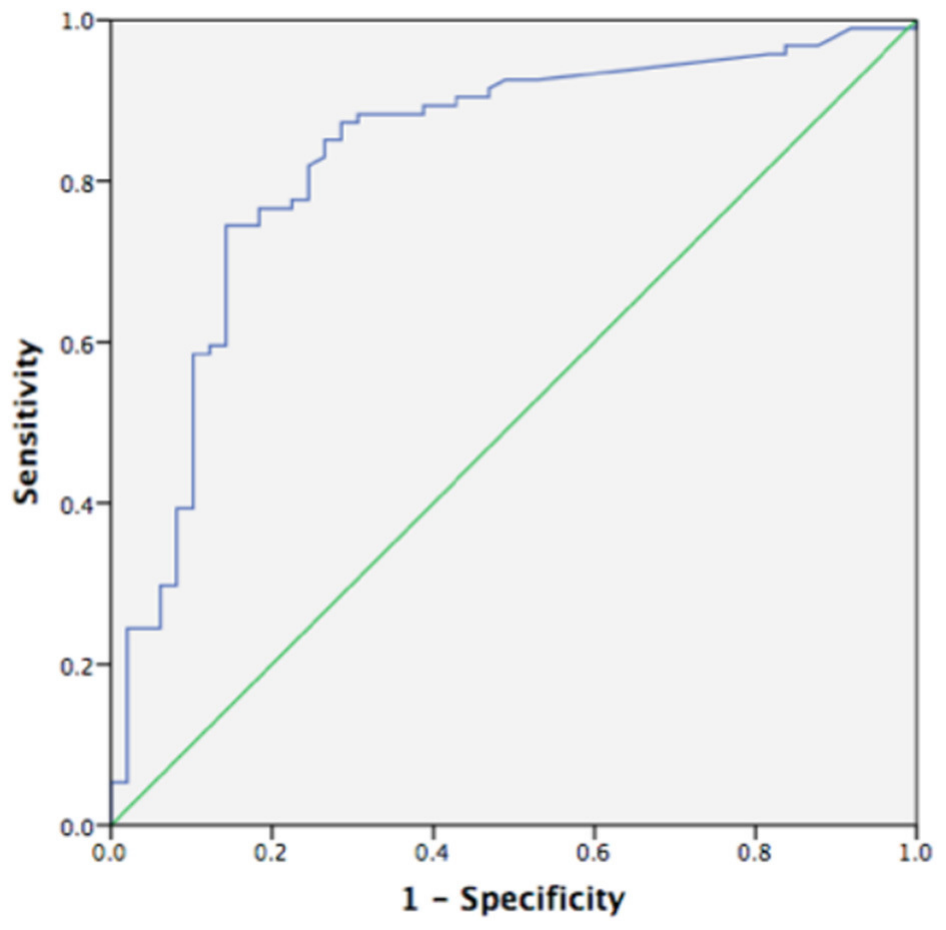
Receiver operating curve analysis (ROC) of fecal calprotectin in detecting clinical activity (AUC 0.830, *p* < 0.05, 95%CI 0.755~0.904).

**Figure 5:**
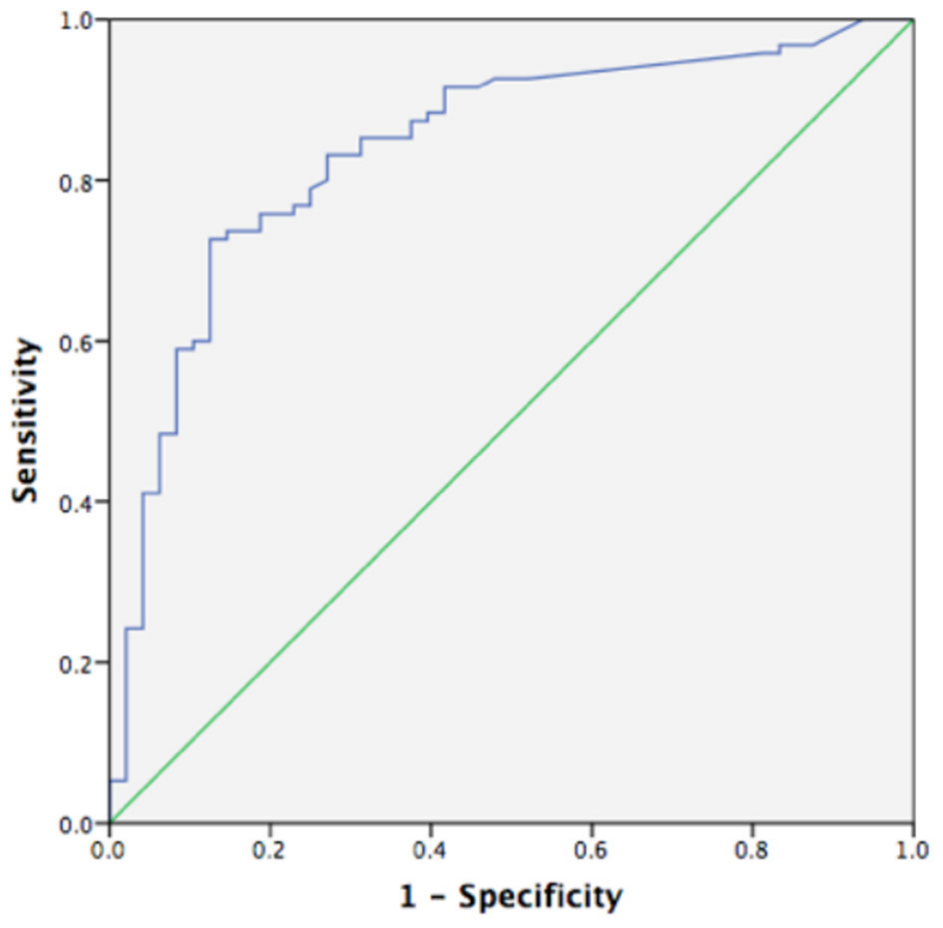
Receiver operating curve analysis (ROC) of fecal calprotectin in MH (AUC 0.839, *p* < 0.05, 95%CI 0.769~0.909).

Using a ROC curve, we attempted to determine the best cut-off value of FC to detect clinical activity and MH. The area under the ROC curve to predict clinical activity and MH was 0.830 and 0.839, respectively (Figures 4, 5). The best cut-off point to detect clinical activity was 164 μg/g (sensitivity 85.42%, specificity 73.68%, PPV 62.12%, NPV 9.10%, AR 77.62%). A cut-off value of 154.5 μg/g indicated MH, with sensitivity of 72.34%, specificity of 85.71%, PPV 90.67%, NPV 38.24%, and AR 76.92%.

## Discussion

In the present study, we assessed the correlation between fecal calprotectin level and clinical/endoscopic scores in UC and showed the performances of FC in detecting clinical activity and endoscopic mucosal healing.

Fecal calprotectin is an abundant protein in neutrophils, which infiltrates the mucosa during inflammation. Data support its use in differentiating inflammatory bowel disease (IBD) from irritable bowel syndrome (IBS) (17–20), evaluating abdominal discomfort (21). Several reports have shown that FC level correlates well with clinical, endoscopic, and histological parameters of disease activity (6, 19) in UC patients. To some extent, FC may reflect disease activity in UC better than in CD as some authors reported (22). FC determination may also be useful in predicting impending clinical relapse especially during the following 3 months in both CD and UC patients (23). FC is also useful in assessing treatment response (24–26).

In the management of patients with UC, endoscopy has an essential role in viewing and evaluating the severity of disease activity in the intestinal mucosa as well as assessing the efficacy of treatment modalities. However, discordance in clinical manifestations and endoscopic findings is not rare. Clinical indices are not reliable in assessing endoscopic MH and in predicting the disease course (27, 28). Evolving evidence indicates that MH is associated with lower risk of long-term complications (29–31). Therefore, currently, MH is of great interest to gastroenterologists and considered as an ideal therapeutic target. However, the exact definition of MH continues to be controversial and several scoring systems have been developed. In our study, we applied UCEIS to define MH as the remission stratum that corresponds to UCEIS 0 or 1. Further, we limited the UCEIS score 1 to a vascular pattern descriptor, so that score 1 of the bleeding descriptor and score 1 of the erosions and ulcers descriptor do not mean real MH. Arai et al. (32) recently reported that UCEIS is useful to predict clinical outcomes and long-term prognosis in UC patients with clinical remission. Consequently, FC had a good correlation with UCEIS. Additionally, we suggest that a definition of MH based on the UCEIS scores may be more relevant.

A recent systematic review (33) showed that fecal markers like FC are promising non-invasive indicators of MH. It is imperative that non-invasive markers become available for routine clinical use. In other words, this could allow more regular assessment of inflammation with subsequent timely clinical decisions and possibly lead to a reduced requirement for follow-up endoscopies. Schoepfer's study (7), the largest study so far, described the diagnostic efficiency of FC to predict mucosal inflammation with sensitivity 93%, specificity 71%, PPV 91%, and NPV 81% using a cut-off 50 μg/g. Yamaguchi et al. (34) analyzed the correlation between FC with both Mayo endoscopic subscore 0 or Mayo endoscopic subscore 0 and 1 defining MH. Not surprisingly, specificity and PPV were greater when using the Mayo 0 score. Based on the interpretations of the ROC graphs, using UCEIS defining MH, we obtained a cut-off FC level of 154.5 μg/g to predict MH with sensitivity 72.34%, specificity 85.71%, and PPV 90.67%. It is not surprising that there has been no agreement regarding an appropriate cut-off level for FC to predict MH (35). Our results are reasonably comparable with these previously published data.

Our sample size could be considered as a limitation of our study. Second, using FC as a predictive tool for MH requires analysis from clinically quiescent patients. This is the biggest weaknesses in our study. Third, the FC levels have also been shown to be variable (36), to overcome this problem we ensured that all patients provided stool samples at least 1 week post biologic administrations. Combination of clinical symptoms and serum and fecal biomarkers is likely to be superior to one single parameter. Such analyses will require well-powered and multicenter studies.

In conclusion, fecal calprotectin could reflect the disease activity of UC and are rational fecal markers of intestinal inflammation for clinical application. FC is also a clinically relevant biomarker of MH in patients with UC, but the value of the cut-off still needs large and multicenter studies for confirmation.

## Data Availability Statement

The raw data supporting the conclusions of this article will be made available by the authors, without undue reservation.

## Ethics Statement

The studies involving human participants were reviewed and approved by Ethics Committee of the First Affiliated Hospital of Zhejiang University of Traditional Chinese Medicine. The patients/participants provided their written informed consent to participate in this study.

## Author Contributions

Y-HF designed the report. FC and YH collected the clinical data. BL contributed to revising the manuscript. FC wrote the manuscript. All authors contributed to the article and approved the submitted version.

## Conflict of Interest

The content of this manuscript has been presented in part at the IBD 2017–Therapeutic and Biological Barriers at Symposium 209 (Berlin) in October 2017. The authors declare that the research was conducted in the absence of any commercial or financial relationships that could be construed as a potential conflict of interest.

## Publisher's Note

All claims expressed in this article are solely those of the authors and do not necessarily represent those of their affiliated organizations, or those of the publisher, the editors and the reviewers. Any product that may be evaluated in this article, or claim that may be made by its manufacturer, is not guaranteed or endorsed by the publisher.
